# Long-term outcome in early survivors of cardiogenic shock at the acute stage of myocardial infarction: a landmark analysis from the French registry of Acute ST-elevation and non-ST-elevation Myocardial Infarction (FAST-MI) Registry

**DOI:** 10.1186/s13054-014-0516-y

**Published:** 2014-09-19

**Authors:** Nadia Aissaoui, Etienne Puymirat, Tabassome Simon, Eric Bonnefoy-Cudraz, Denis Angoulvant, Francois Schiele, Hakim Benamer, Philippe Quandalle, Fabrice Prunier, Eric Durand, Laurence Berard, Didier Blanchard, Nicolas Danchin

**Affiliations:** Department of Cardiology, Assistance Publique-Hôpitaux de Paris, Hôpital Européen Georges Pompidou, Paris, France; Faculty of Medicine, University Paris Descartes, Paris, France; INSERM U970, Paris Cardiovascular Research Center PARCC, Paris, France; Assistance Publique-Hôpitaux de Paris,CHU St Antoine, Paris, France; INSERM U-698, Paris, France; UPMC, Paris 06, Paris, France; Intensive Cardiac Care Unit Hôpital Cardio-pneumologique, Lyon, France; Department of Cardiology CHRU de Tours and EA 4245 Faculty of Medicine, University François Rabelais, Tours, France; CHU Besancon, Besancon, France; Hôpital Privé Jacques Cartier, Massy, France; Hôpital Victor Provo, Roubaix, France; Department of Cardiology, CHU Angers, and l’UNAM Université, Laboratoire Cardioprotection Remodelage et Thrombose, Angers, France; CHU de Rouen, Rouen, France; Clinique St Gatien, Tours, France

## Abstract

**Introduction:**

There are little data about patients with cardiogenic shock (CS) who survive the early phase of acute myocardial infarction (AMI). The aim of this study was to assess long-term (5-year) mortality among early survivors of AMI, according to the presence of CS at the acute stage.

**Methods:**

We analyzed 5-year follow-up data from the French registry of Acute ST-elevation and non-ST-elevation Myocardial Infarction (FAST-MI) 2005 registry, a nationwide French survey including consecutive patients admitted for ST or non-ST-elevation AMI at the end of 2005 in 223 institutions.

**Results:**

Of 3670 patients enrolled, shock occurred in 224 (6.1%), and 3411 survived beyond 30 days or hospital discharge, including 99 (2.9%) with shock. Early survivors with CS had a more severe clinical profile, more frequent concomitant in-hospital complications, and were less often managed invasively than those without CS.

Five-year survival was 59% in patients with, versus 76% in those without shock (adjusted hazard ratio (HR) = 1.72 [1.24-2.38], *P* = 0.001). The excess of death associated with CS, however, was observed only during the first year (one-year survival: 77% vs 93%, adjusted HR: 2.87 [1.85 to 4.46] *P* <0.001), while survival from one to 5 years was similar (76% vs 82%, adjusted HR: 1.06 [0.64 to 1.74]). Propensity score-matched analyses yielded similar results.

**Conclusions:**

In patients surviving the early phase of AMI, CS at the initial stage carries an increased risk of death up to one year after the acute event. Beyond one year, however, mortality is similar to that of patients without shock.

**Trial registration:**

ClinicalTrials.gov number, NCT00673036, Registered May 5, 2008.

**Electronic supplementary material:**

The online version of this article (doi:10.1186/s13054-014-0516-y) contains supplementary material, which is available to authorized users.

## Introduction

Despite considerable progress in the management of patients admitted for acute myocardial infarction (AMI), cardiogenic shock (CS) remains a major complication [1,2]. In-hospital mortality has declined due to early revascularization and improved overall management, however, early mortality rates remain high; only about 50% of AMI patients developing CS are alive at one month [1-4].

Little data exist on the long-term outcomes of patients surviving the acute phase of CS following AMI [4-6]. Most studies report that patients with CS have an increased risk of death up to one year after the acute event. Beyond 1 year, mortality seems to be similar to that of patients without CS. However, most of these studies were performed in the 1990s., Marked reductions in AMI mortality have recently been reported, including among patients with CS [4-6].

The French registry of Acute ST-elevation and non-ST-elevation Myocardial Infarction (FAST MI) 2005 registry is a prospective, nationwide, observational study conducted at the end of 2005 in a large number of the French hospitals treating AMI patients [7,8]. It allows evaluation of the long-term outcome in acute ST-elevation and non-ST-elevation myocardial infarction patients with and without CS. The aim of the present study was to analyse long-term outcomes of early survivors of the acute phase, according to the presence of CS at the acute stage.

## Materials and methods

### Study population

The methods of the FAST-MI 2005 registry have been described in detail elsewhere [7,8]. Briefly, the primary objectives were to evaluate myocardial infarction (MI) management in real-life practice and to assess short- and long-term outcomes of patients admitted to ICUs for MI. Patients were consecutively recruited from ICU departments over a period of 1 month (October to November 2005), with a 1-month extension for patients with known diabetes mellitus. Participation in the study was offered to all French institutions, university teaching hospitals, general and regional hospitals, and private clinics with ICUs authorized to receive acute coronary syndromes (ACS). In each centre, a physician was in charge of the registry and provided a full list of all patients admitted to the unit. The number of participating centres was 223, representing 60% of all centres taking care of AMI patients in France at that time.

Inclusion criteria were (1) men or women, over 18 years old; (2) patients admitted within 48 h after symptom onset in an ICU for an AMI characterized by increased troponin or creatine kinase-MB associated with at least one of the following elements: symptoms compatible with myocardial ischaemia, appearance of pathologic Q-waves, or ST-T changes compatible with myocardial ischaemia (ST-segment elevation or depression, T-wave inversion); and (3) consent to take part in the study. Patients who died very soon after admission and for whom cardiac markers were not measured were included if they had compatible signs or symptoms associated with typical ST-segment changes. Exclusion criteria were (1) refusal to participate; (2) patients with MI who were admitted more than 48 h after symptom onset; (3) patients with iatrogenic MI, defined as MI occurring within 48 h of a therapeutic procedure (bypass surgery, coronary angioplasty, or any other medical or surgical intervention); (4) ACS diagnosis invalidated in favour of another diagnosis; and (5) patients with unstable angina and no increase in cardiac biomarkers.

CS was defined as systolic blood pressure <90 mm Hg for ≥1 h not responsive to fluid administration alone, thought to be secondary to cardiac dysfunction, and associated with signs of hypoperfusion or cardiac index ≤2.2 l/min/mm2 and pulmonary capillary wedge pressure >18 mm Hg [9,10].

All patients provided informed consent for their participation in the registry. The protocol was reviewed by the Committee for the Protection of Human Subjects in Biomedical Research of St Antoine University Hospital and the data file of the study was declared to and authorized by the French data protection committee (*Commission Nationale Informatique et Liberté*). Participating physicians were asked not change their usual therapeutic approach for the purpose of the survey. All the authors vouch for the fidelity of the study to the trial protocol, which is available at ClinicalTrials.gov number, NCT00673036 (Registered 5 May 2008).

Overall, 3,670 patients were included in the survey. Among them, 224 (6.1%) developed shock: 259 patients died in hospital or during the first 30 days following admission, and 3,411 patients survived the early phase (patients surviving the initial hospital stay and alive at 30 days) and were included in the present study.

### Data collection

Baseline characteristics, namely demographics (age, gender), risk factors (hypertension, body mass index >30 kg/m^2^, diabetes, current smoking, hyperlipidaemia, family history), medical history (previous AMI, previous percutaneous coronary intervention (PCI), previous coronary artery bypass grafting, previous stroke, previous heart failure, prior peripheral arterial disease, previous chronic renal failure, previous chronic obstructive pulmonary disease and previous cancer), and previous medications (antiplatelet agents, statins, angiotensin-converting enzyme (ACE) inhibitors, angiotensin-receptor blockers (ARB)s, beta-blockers and insulin) were collected prospectively and stored electronically as previously described.

Clinical presentation, and glycaemia at the time of admission were also collected and the last value of left ventricular ejection fraction (LVEF) during the hospital stay was recorded. We also recorded the use of cardiac procedures, in-hospital complications (re-infarction, stroke, major bleeding, the need for transfusion, ventricular fibrillation, new atrial fibrillation and second-and third-degree atrio-ventricular (AV) block) and medications (antiplatelet agents, diuretics, beta-blockers, ACE-inhibitors and lipid-lowering agents) used in the first 48 h and at hospital discharge in early CS survivors.

### Outcome

Mortality was assessed at 1 and 5 years both in patients with and without CS, who were discharged alive and were alive at one month (early survivors). Follow up was centralised at the French Society of Cardiology and dedicated research technicians contacted both physicians and patients themselves, after checking patients’ vital status in municipal registries. Causes of death were obtained by direct contact with patients’ physicians or families, and from the national causes of death registry. Two cardiologists, unaware of patients’ hospital course, adjudicated causes of death as cardiovascular, non-cardiovascular, or unknown. The rate of patients lost to follow up was 0.3% at 1 year, 2% at 3 years and 5% at 5 years.

### Statistical analysis

Statistical analysis was performed using IBM SPSS 20.0 (IBM SPSS, Inc., Armonk, NY, USA) and NCSS 9.0.7 (NCSS, LLC. Kaysville, UT, USA). For quantitative variables, mean and standard deviations were calculated. Discrete variables are presented as percentages. Comparisons were performed with the chi-square or Fisher’s exact test for discrete variables and with the unpaired *t*-test, or Wilcoxon sign-rank tests for continuous variables. Odds ratios (OR) or hazard ratios (HR) are presented with the 95% CI. Five-year mortality rates were calculated using the Kaplan-Meier method and comparisons were made using log-rank tests.

Because coronary artery disease remains unstable for several months after an acute coronary syndrome, and potent antithrombotic medications, such as dual antiplatelet therapy, are recommended for 1 year following AMI, we selected the 1-year time point for performing landmark analyses: the analyses were performed with 1-year survival as the dependent variable in the population of early survivors, and 5-year survival as the dependent variable in the population of patients alive at one year [11].

Correlates of 5-year mortality were determined using a multivariate Cox backward analysis. The cumulative hazard functions for each covariable were computed to assess proportionality, and colinearity was verified by calculating variance inflation factors. Shock was analysed as a time-dependent variable, with the time-point set at 12 months from the acute episode. Variables included in the final multivariate models were selected ad hoc, based upon their physiological relevance and potential to be associated with outcomes; thus, we included variables likely to influence outcome negatively (age, history of heart failure, history of diabetes, history of prior AMI, history of stroke, history of peripheral artery disease, anemia on admission) or positively (history of hypertension, current smoking, revascularisation by percutaneous coronary intervention or surgery, early use of low molecular weight heparin, glycoprotein IIB-IIIa inhibitors, and discharge medications: aspirin, clopidogrel, statins, ACE-inhibitors, ARBs, beta-blockers) as well as sex, type and region of institution, and type of MI (segment-elevation myocardial infarction (STEMI) versus non-segment-elevation myocardial infarction (NSTEMI).

In addition, to assess the potential role of CS on late mortality, we calculated propensity scores for having presented CS, using logistic regression analysis 1) in patients alive at hospital discharge and at 30 days, and 2) in patients who were alive at one year (*c*-statistic = 0.76 for both propensity scores). Cohorts with and without CS were constituted, matched on the propensity scores (3-to-1 matching: population of early survivors, 94 patients with CS, 282 patients without CS; population of 1-year survivors, 73 patients with CS, 217 patients without CS), and their outcomes were compared using log-rank tests. For calculating the propensity scores, we used baseline characteristics, early management including revascularisation procedures and antithrombotic medications, in-hospital complications (re-infarction and major bleeding) and discharge medications. LVEF and medications indicated for heart failure (renin angiotensin aldosterone inhibitors and beta-blockers) were not included in the model to avoid over-adjustment. For all analyses, a *P*-value <0.05 was considered significant.

## Results

### Baseline characteristics of all 30-day survivors

Among the 3,411 early survivors, 99 (2.9%) had developed CS at the acute stage. Patients with CS were significantly older and had more comorbid conditions (Table 1). Clinical presentation at admission of patients with CS was more severe, and they had developed more complications during hospitalization. The use of revascularization procedures was comparable in the two groups.

**Table 1:** **Baseline characteristics of patients surviving at 30 days and hospital discharge according to presence of cardiogenic shock at the acute stage**

	**No shock (n = 3,312)**	**Shock (n = 99)**	***P*** **-value**
Age, years, mean ± SD	66 ± 14	70 ± 13	<0.001
Sex, female, n (%)	1014 (30.6)	37 (37.4)	0.43
Body mass index, Kg/m^2^, mean ± SD	27.2 ± 4.7	26.5 ± 4.9	0.15
**Risk factors, n (%)**			
Hypertension	1949 (58.8)	62 (62.6)	0.45
Diabetes mellitus	1166 (35.2)	37 (37.4)	0.65
Current smoking	990 (29.9)	27 (27.3)	0.59
Hypercholesterolemia	1622 (49.0)	44 (44.4)	0.37
Family history of coronary artery disease	796 (24.0)	11 (11.1)	0.003
**Previous medical history, n (%)**			
Myocardial infarction	591 (17.8)	23 (23.2)	0.17
Percutaneous coronary intervention	472 (14.3)	13 (13.1)	0.75
Coronary artery bypass graft	186 (5.6)	8 (8.1)	0.30
Stroke	168 (5.1)	5 (5.1)	0.99
Peripheral arterial disease	316 (9.6)	17 (17.2)	0.01
Heart failure	172 (5.2)	9 (9.1)	0.09
Chronic kidney disease	172 (5.2)	6 (6.1)	0.71
Chronic obstructive pulmonary disease	141 (4.3)	9 (9.1)	0.02
Cancer	218 (6.6)	4 (4.0)	0.31
**Previous medications, n (%)**			
Antiplatelet agents	1058 (31.9)	35 (35.4)	0.47
Statins	930 (28.1)	32 (32.4)	0.35
Angiotensin-converting enzyme inhibitor	640 (19.3)	33 (33.3)	0.001
Angiotensin receptor blockers	515 (15.5)	13 (13.1)	0.52
Beta-blockers	828 (25.0)	28 (28.3)	0.46
Insulin	335 (10.1)	13 (13.1)	0.33
**Current episode, n (%)**			
Typical chest pain	2538 (79.0)	58 (65.2)	0.002
	(n = 3213)	(n = 89)	
Resuscitated cardiac arrest	31 (0.9)	10 (10.1)	<0.001
Segment-elevation myocardial infarction	1688 (50.9)	54 (54.5)	0.47
Anaemia on admission	689 (21.5) (n = 3202)	35 (36.8) (n = 95)	<0.001
Admission glycaemia, mg/dl, mean ± SD	156 ± 77	203 ± 109	<0.001
Left ventricular ejection fraction, %, mean ± SD	53 ± 13	42 ± 16	<0.001
**Medications within the first 48 h, n (%)**			
Low molecular-weight heparin	2159 (65.1)	45 (45.5)	<0.001
Clopidogrel	2882 (86.9)	81 (81.8)	0.14
GP IIb-IIIa inhibitors	1236 (37.3)	35 (35.4)	0.69
**Procedures during hospital stay, n (%)**			
Coronary angiography	2898 (87.4)	79 (79.8)	0.03
Percutaneous coronary intervention	2186 (65.9)	60 (60.9)	0.27
Coronary artery bypass graft	140 (4.2)	4 (4.0)	0.93
**In-hospital complications, n (%)**			
Re-infarction	51 (1.5)	3 (3.0)	0.24
Stroke	19 (0.6)	4 (4.0)	<0.001
Major bleeding	54 (1.6)	7 (7.1)	<0.001
Transfusion	119 (3.6)	13 (13.1)	<0.001
Ventricular fibrillation	44 (1.3)	12 (12.1)	<0.001
Atrial fibrillation, new	150 (4.5)	25 (25.3)	<0.001
Atrio-ventricular block	39 (1.2)	4 (4.0)	0.01
**Medications at discharge, n (%)**			
Aspirin	3039 (92.3)	88 (90.7)	0.58
Clopidogrel	2662 (81.0)	78 (7906)	0.73
Statins	2760 (84.4)	70 (72.9)	0.002
Beta-blockers	2566 (78.7)	62 (65.3)	0.002
Angiotensin-converting enzyme inhibitors	2030 (62.9)	66 (68.8)	0.24
Angiotensin receptor blockers	270 (8.6)	4 (4.3)	0.15
Aldosterone receptor blockers	153 (4.9)	17 (18.3)	<0.001
Loop diuretics	613 (19.7)	53 (57.6)	<0.001
Digoxin	17 (0.5)	0	0.48
Nitrates	581 (18.6)	25 (27.2)	0.04
Amiodarone	232 (7.0)	24 (24.2)	<0.001

### Five-year survival

**Figure 1:**
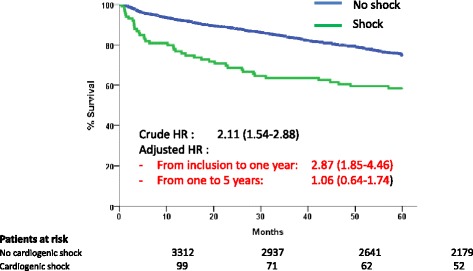
**Five-year survival in patients surviving at 30 days and hospital discharge according to the cardiogenic shock status.** HR, hazard ratio.

Five-year survival was 59% in early survivors with CS, compared with 76% in early survivors without shock (*P* <0.001) (Figure 1).

### Landmark analyses in the overall population

One-year survival was 93% in early survivors without CS and 77% in patients with CS. Cause of death analysis showed that cardiovascular death was as frequent in CS patients (65.2%) as in non-CS patients (66.3%). Five-year survival was 76% in the 76 CS patients surviving at one year, versus 82% in the 3,072 non-CS patients surviving at one year (an additional table shows this in more detail; see Additional file 1). Beyond 1 year, there was a non-significant trend towards increased cardiovascular mortality in CS patients (55.6% versus 36.3%), but with fewer deaths of unknown cause (22.2% versus 31.1%).

In the time-dependent Cox multivariate model, CS was independently associated with increased hazard for death at 12 months (HR: 2.87, 95% CI: 1.85, 4.46, *P* <0.001), but carried no increased risk from 1 year to 5 years (HR 1.06, 95% CI: 0.64, 1.74).

### Propensity-score-matched population

**Figure 2:**
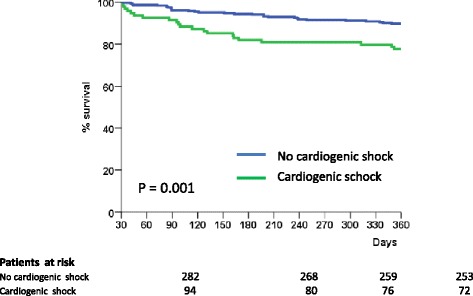
**Landmark analysis: 1-year survival in propensity-score-matched cohorts of early survivors.**

Separate analyses on propensity-score-matched populations confirmed these data: 1-year mortality in propensity-score-matched cohorts of early survivors was significantly higher in those with CS at the acute stage (HR: 2.49, 95% CI: 1.43, 4.33, *P* = 0.001) (Figure 2 and Additional file 2 show this in more detail).

**Figure 3:**
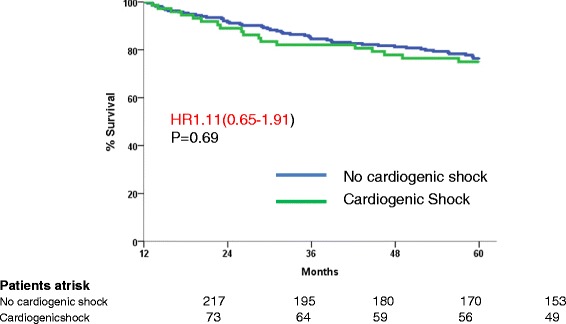
**Landmark analysis: from one year to five years.** Five-year survival in propensity-score-matched cohorts of patients surviving at one year. HR, hazard ratio.

In contrast, 5-year mortality in propensity-score-matched cohorts of 1-year survivors was similar in patients with CS at the acute stage (25%) and in those without (23%) (HR: 1.11, 95% CI: 0.65, 1.91, *P* = 0.69) (Figure 3 and Additional file 3 show this in more detail). In this population, similar characteristics were correlated with long-term survival in patients with or without CS (see Additional file 4).

### Predictors of 1-year death in early survivors with shock

Using Cox multivariate analysis in the population of patients with CS, most prognostic factors for death at 1 year were un-modifiable (older age, diabetes mellitus, history of chronic kidney disease); likewise, patients with STEMI had a markedly increased risk, compared with NSTEMI patients (HR: 4.31; 95% CI 1.38, 13.44). When STEMI patients were categorised according to use of reperfusion therapy at the acute stage, and compared with NSTEMI patients, lack of reperfusion in STEMI patients was related to poorer survival (HR: 6.44, 95% CI: 1.91, 21.7 versus NSTEMI), while only a trend persisted for STEMI patients with reperfusion therapy (HR: 2.87, 95% CI: 0.69, 11.9 versus NSTEMI). None of the discharge medications were associated with improved survival, although non-significant trends were observed for statins (HR: 0.43, 95% CI: 0.10, 1.75) and clopidogrel (HR: 0.37, 95% CI: 0.08, 1.60).

### Predictors of 5-year death in 1-year survivors with shock

In 1-year survivors who had CS at the acute stage, both older age, and previous history of coronary artery disease were associated with lower survival rate. Revascularisation procedures during the initial hospital stay were associated with lower 5-year mortality (HR: 0.12, 95% CI: 0.03, 0.42).

## Discussion

The main finding of the present study in a contemporary real-world population of AMI patients, is that early survivors of CS still have higher mortality at 1 year, compared with early survivors without CS. Beyond 1 year, however, mortality up to 5 years becomes comparable between patients with or without CS at the acute stage, although the former have a more severe clinical profile. Mortality is higher in STEMI patients with CS than in NSTEMI patients with CS, and early revascularization is associated with better long-term survival.

Most data available on the long-term outcome of CS patients surviving the early phase of AMI were obtained before major changes in AMI treatment were widely implemented [3-5]. They are derived from populations selected for inclusion into randomized trials, or less frequently from real-life populations. Patient inclusion was usually before the year 2000, at a time when outcomes were notably poorer than nowadays [12].

Despite improved early management, early mortality in CS patients remains considerably higher than that of patients without CS [2,13]. In prior studies the reported overall long-term survival of CS patients surviving the early period varies widely from 12% to 73% at 5 years [4-6,14,15]. In the large GUSTO-1 population of STEMI patients [5], mortality at 11 years in early survivors was 45% in patients with CS, compared with 31% in patients without CS. Patients included in the trial were relatively young and had to fulfill the trial inclusion criteria, which included early presentation after symptom onset, and all received intravenous fibrinolytic therapy. Our patients were enrolled in 2005, and more than 80% of patients underwent myocardial revascularization. This clearly differentiates our study, which is therefore clinically relevant in the current era.

To the best of our knowledge, there has been only one recent report on a large contemporary series of patients with long-term follow up after CS [16]. The data, gathered from the CRUSADE registry cross-linked with the Medicare administrative database, were limited to older (≥65 years) patients and only included NSTEMI patients. Four-year survival in CS early survivors was 48%, compared with 56.5% in non-CS survivors. Another novelty of our study is that both STEMI and NSTEMI patients were included.

In this population, 5-year survival of CS patients alive after the acute phase was 59%. Improvements in primary angioplasty and adjunctive pharmacotherapy are likely to explain higher survival rates [4,5] in recent series. Of note, a majority of our patients had undergone myocardial revascularization during the initial hospital stay and early revascularization was associated with better long-term survival. In the SHOCK trial, early revascularization significantly reduced 6-year mortality in early survivors by 41% (absolute risk reduction: 18%) [4]. Our study underlines the potential interest of performing immediate coronary reperfusion in CS, as it is also associated with improved long-term survival. In this regard, developing networks for the management of AMI patients, particularly for the most severely affected patients, seems important to improve both early and long-term outcomes.

A consistent finding in all studies, including ours, is similar mortality beyond 1 year in patients with or without CS, regardless of the period studied or the inclusion of STEMI or NSTEMI patients. The GUSTO-1 study confirmed similar long-term survival between CS and non-CS patients who were alive at 1 year. In the much more recent CRUSADE registry in NSTEMI patients 65 years of age or older, no difference was observed between patients with or without CS, once they had survived the first months following the acute episode [16]. The reasons for late survival becoming similar are unclear, especially when considering the difference in LVEF between patients who have developed CS and those who have not. Mechanisms leading to CS include left ventricular dysfunction, systemic inflammatory response, activation of complement, release of cytokines, and expression of inducible nitric oxide synthase [4,14,17]. The recent IABP SHOCK II trial confirmed the presence of a high degree of inflammatory response in CS patients, irrespective of the management strategy, and that CS could be present even in the absence of profound LV dysfunction (median LVEF 35%) [17]. The resolution of severe ischaemia and/or neurohormonal abnormalities may explain the potential reversibility of shock [4,14]. Also, the increasing use of medications such as beta-blockers, ACE-inhibitors, ARBs or aldosterone blockers in patients with CS during the hospital admission may have participated in their improved long-term survival; of note, the percentage of patients with CS receiving beta-blockers increased from 68% at discharge to 81% at one year, and the percentage of patients receiving either ACE-inhibitors or ARBs marginally increased from 78% to 81% .

### Strengths and limitations

Our study has the usual limitation of observational data. Namely, no causality can be inferred from the associations we observed. In addition, the sample size of patients with CS was small, and subgroup analyses were therefore unrealistic. We excluded patients admitted more than 48 h after symptoms onset. These patients could have developed CS. Likewise, we were not able to assess the potential effect of implantable cardioverter defibrillators or of cardiac resynchronization therapy, as very few patients were implanted during the initial hospital stay (one in the CS group and six in the patients without CS). Conversely, our population was extremely well-characterized and reflected a real-world population. Also, it included both STEMI and NSTEMI patients and the rate of patients lost to follow up was low.

## Conclusion

The long-term outcome for early survivors of CS is worse than that of patients without CS. Patients who survive the first year after the acute event, however, have a 5-year survival rate similar to that of non-CS patients. This divergence between early, semi-early, and long-term outcomes has remained consistent since the late 1980s and underlines the importance of improving short-term outcome by the early detection and management of CS, including early myocardial revascularisation.

## Key messages

- Early survivors of CS still have a higher mortality at 1 year, compared with early survivors without CS
- Beyond 1 year, however, mortality up to 5 years becomes comparable between patients with or without CS at the acute stage, although the former have a more severe initial profile
- Mortality is higher in STEMI patients with CS than in NSTEMI patients with CS
- Early revascularization is associated with better long-term survival in CS patients following acute myocardial infarction

## Additional files

Additional file 1:
**Baseline, in-hospital and 1-year characteristics of the patients alive at one year.**
Additional file 2:
**Propensity-score-matched cohorts of patients alive at hospital discharge and 30 days.**
Additional file 3:
**Propensity-score-matched cohorts of patients alive at one year. **
Additional file 4:
**Characteristics of patients in the propensity-score-matched cohorts of 1-year survivors according to survival status at 5 years.**

## Abbreviations

ACE: angiotensin-converting enzyme
AMI: acute myocardial infarction
ARB: angiotensin-receptor blocker
AV: atrio-ventricular
CS: cardiogenic shock
FAST-MI: French registry of Acute ST-elevation and non-ST-elevation Myocardial Infarction
LVEF: left ventricular ejection fraction
MI: myocardial infarction
NSTEMI: non-segment-elevation myocardial infarction
STEMI: segment-elevation myocardial infarction
