# Supplementary material for: Long-term outcome in early survivors of cardiogenic shock at the acute stage of myocardial infarction: a landmark analysis from the French registry of Acute ST-elevation and non-ST-elevation Myocardial Infarction (FAST-MI) Registry
Source: Crit Care. 2014 Sep 19;18(5):516. doi: 10.1186/s13054-014-0516-y (PMC4192440; doi:10.1186/s13054-014-0516-y)
Supplement: Additional file 1: — Baseline, in-hospital and 1-year characteristics of the patients alive at one year. [file 13054_2014_516_MOESM1_ESM.doc]

**Additional file 1: Table S1: Baseline, in-hospital and one-year characteristics of the patients alive at one year**

|  | **No shock**  **N=3072** | **Shock**  **N=76** | **P value** |
| --- | --- | --- | --- |
| Age (years), mean ± SD | 65 ± 14 | 68 ± 13 | 0.08 |
| Sex (F) | 915 (29.8) | 24 (31.6) | 0.74 |
| BMI (Kg/m²) mean ± SD | 27.4 ± 4.7 | 26.7 ± 4.9 | 0.27 |
|  |  |  |  |
| **Risk factors** |  |  |  |
| Hypertension | 1759 (57.3) | 41 (53.9) | 0.56 |
| Diabetes mellitus | 1040 (33.9) | 24 (31.6) | 0.68 |
| Current smoking | 964 (31.4) | 22 (28.9) | 0.66 |
| Hypercholesterolemia | 1514 (49.3) | 35 (46.1) | 0.57 |
| Family history of CAD | 775 (25.2) | 7 (9.2) | 0.001 |
|  |  |  |  |
| **Previous medical history** |  |  |  |
| Myocardial infarction | 515 (16.8) | 19 (25.0) | 0.059 |
| PCI | 439 (14.3) | 10 (13.2) | 0.78 |
| CABG | 158 (5.1) | 6 (7.9) | 0.29 |
| Stroke | 135 (4.4) | 3 (3.9) | 0.85 |
| Peripheral arterial disease | 266 (8.7) | 11 (14.5) | 0.08 |
| Heart failure | 125 (4.1) | 7 (9.2) | 0.03 |
| Chronic kidney disease | 133 (4.3) | 2 (2.6) | 0.47 |
| COPD | 116 (3.8) | 6 (7.9) | 0.07 |
| Cancer | 175 (5.7) | 2 (2.6) | 0.25 |
|  |  |  |  |
| **Previous medications** |  |  |  |
| Antiplatelet agents | 939 (30.6) | 23 (30.3) | 0.95 |
| Statins | 851 (27.7) | 25 (32.9) | 0.32 |
| ACE-inhibitors | 568 (18.5) | 24 (31.6) | 0.004 |
| ARBs | 472 (15.4) | 10 (13.2) | 0.60 |
| Beta-blockers | 754 (24.5) | 18 (23.7) | 0.86 |
| Insulin | 280 (9.1) | 9 (11.8) | 0.42 |
|  |  |  |  |
| **Current episode** |  |  |  |
| Typical chest pain | 2392 (80.1) n=2985 | 46 (66.7) n=69 | 0.006 |
| Resuscitated cardiac arrest | 29 (0.9) | 8 (10.5) | <0.001 |
| ST-elevation MI | 1614 (52.5) | 39 (51.3) | 0.83 |
| Anemia on admission | 581 (19.6) | 22 (30.6) | 0.02 |
| Admission glycemia (mg/dl) mean ± SD | 154 ± 73 | 187 ± 99 | <0.001 |
| LVEF (%) mean ± SD | 53 ± 12 | 44 ± 16 | <0.001 |
|  |  |  |  |
| **Medications within first 48 hours** |  |  |  |
| Low molecular weight heparin | 2041 (66.4) | 37 (48.7) | 0.001 |
| Clopidogrel | 2714 (88.3) | 83 (82.9) | 0.14 |
| GP IIb-IIIa inhibitors | 1197 (39.0) | 29 (38.2) | 0.88 |
|  |  |  |  |
| **Procedures during hospital stay** |  |  |  |
| Coronary angiography | 2777 (90.4) | 63 (82.9) | 0.03 |
| PCI | 2103 (68.5) | 48 (63.2) | 0.33 |
| CABG | 138 (4.5) | 4 (5.3) | 0.75 |
|  |  |  |  |
| **In-hospital complications** |  |  |  |
| Reinfarction | 41 (1.3) | 2 (2.6) | 0.34 |
| Stroke | 16 (0.5) | 3 (3.9) | <0.001 |
| Major bleeding | 43 (1.4) | 6 (7.9) | <0.001 |
| Transfusion | 97 (3.2) | 9 (11.8) | <0.001 |
| Ventricular fibrillation | 43 (1.4) | 11 (14.5) | <0.001 |
| Atrial fibrillation (new) | 134 (4.4) | 17 (22.4) | <0.001 |
| AV block | 34 (1.1) | 3 (3.9) | 0.0243 |
|  |  |  |  |
| **Medications at discharge** |  |  |  |
| Aspirin | 2829 (92.6) | 68 (90.7) | 0.52 |
| Clopidogrel | 2516 (82.5) | 59 (78.7) | 0.38 |
| Statin | 2596 (85.5) | 58 (78.4) | 0.09 |
| Beta-blocker | 2419 (79.9) | 50 (68.5) | 0.02 |
| ACE-inhibitor | 1898 (63.4) | 56 (75.7) | 0.03 |
| ARB | 247 (8.5) | 2 (2.9) | 0.09 |
| Aldosterone receptor blocker | 139 (4.8) | 14 (19.7) | <0.001 |
| Loop diuretic | 490 (17.0) | 39 (55.7) | <0.001 |
| Digoxin | 15 (0.5) | 0 | 0.55 |
| Nitrates | 515 (17.7) | 16 (22.9) | 0.27 |
| Amiodarone | 194 (6.3) | 18 (23.7) | <0.001 |
|  |  |  |  |
| **Internal cardioverter defibrillator implanted during the first year** | 19 (0.9) | 5 (5.1) | <0.001 |
|  |  |  |  |
| **Medications at one year** | N=2355 | N=58 |  |
| Aspirin | 1967 (83.5) | 48 (82.8) | 0.88 |
| Clopidogrel | 1699 (72.1) | 38 (65.5) | 0.27 |
| Statin | 1961 (83.3) | 45 (77.6) | 0.25 |
| Beta-blocker | 1837 (78.0) | 47 (81.0) | 0.58 |
| ACE-inhibitor | 1418 (60.2) | 37 (63.8) | 0.58 |
| ARB | 354 (15.0) | 12 (20.7) | 0.23 |
| Aldosterone receptor blocker | 142 (6.0) | 10 (17.0) | 0.001 |

**Abbreviations:** ACE: angiotensin converting enzyme; ARB: angiotensin receptor blockers; AV: atrio-ventricular; BMI: body mass index; CABG: coronary artery bypass graft; CAD: coronary artery disease; COPD: chronic obstructive pulmonary disease; LVEF left ventricular ejection fraction; PCI: percutaneous coronary intervention; STEMI: ST-segment elevation myocardial infarction.
