# Supplementary material for: Long-term outcome in early survivors of cardiogenic shock at the acute stage of myocardial infarction: a landmark analysis from the French registry of Acute ST-elevation and non-ST-elevation Myocardial Infarction (FAST-MI) Registry
Source: Crit Care. 2014 Sep 19;18(5):516. doi: 10.1186/s13054-014-0516-y (PMC4192440; doi:10.1186/s13054-014-0516-y)
Supplement: Additional file 2: — Propensity-score-matched cohorts of patients alive at hospital discharge and 30 days. [file 13054_2014_516_MOESM2_ESM.doc]

**Additional file 2: Table S2: Propensity score-matched cohorts of patients alive at hospital discharge and 30 days**

|  | **No shock**  **N=282** | **Shock**  **N=94** | **P value** |
| --- | --- | --- | --- |
| Age (years), mean ± SD | 70.7 ± 13.4 | 70.3 ± 13.2 | 0.82 |
| Sex (F) | 118 (41.8) | 37 (39.4) | 0.67 |
| BMI (Kg/m²) mean ± SD | 26.8 ± 5.3 | 26.6 ± 4.9 | 0.78 |
|  |  |  |  |
| **Risk factors** |  |  |  |
| Hypertension | 176 (62.4) | 59 (62.8) | 0.95 |
| Diabetes mellitus | 95 (33.7) | 36 (38.3) | 0.42 |
| Current smoking | 74 (26.2) | 24 (25.5) | 0.89 |
| Hypercholesterolemia | 130 (46.1) | 44 (46.8) | 0.90 |
| Family history of CAD | 32 (11.3) | 11 (11.7) | 0.92 |
|  |  |  |  |
| **Previous medical history** |  |  |  |
| Myocardial infarction | 70 (24.8) | 23 (24.5) | 0.94 |
| PCI | 37 (13.1) | 12 (12.8) | 0.93 |
| CABG | 21 (7.4) | 8 (8.5) | 0.74 |
| Stroke | 19 (6.7) | 5 (5.3) | 0.63 |
| Peripheral arterial disease | 42 (14.9) | 15 (16.0) | 0.80 |
| Heart failure | 26 (9.2) | 9 (9.6) | 0.92 |
| Chronic kidney disease | 18 (6.4) | 6 (6.4) | 1.00 |
| COPD | 25 (9.2) | 9 (9.6) | 0.92 |
| Cancer | 10 (3.5) | 4 (4.3) | 0.75 |
|  |  |  |  |
| **Previous medications** |  |  |  |
| Antiplatelet agents | 89 (31.6) | 33 (35.1) | 0.52 |
| Statins | 92 (32.6) | 32 (34.0) | 0.80 |
| ACE-inhibitors | 95 (33.7) | 32 (34.0) | 0.95 |
| ARBs | 41 (14.5) | 13 (13.8) | 0.86 |
| Beta-blockers | 79 (28.0) | 28 (29.8) | 0.74 |
| Insulin | 38 (13.5) | 12 (12.8) | 0.86 |
|  |  |  |  |
| **Current episode** |  |  |  |
| Typical chest pain | 192 (69.8) | 56 (66.7) | 0.58 |
| Resuscitated cardiac arrest | 1 (0.4) | 10 (10.6) | <0.001 |
| ST-elevation MI | 169 (59.9) | 50 (53.2) | 0.25 |
| Anemia on admission | 996 (36.0) | 31 (34.4) | 0.80 |
| Admission glycemia (mg/dl) mean ± SD | 158 ± 80 | 200 ± 108 | <0.001 |
| LVEF (%) mean ± SD | 49 ± 13 | 42 ± 16 | <0.001 |
|  |  |  |  |
| **Medications within the first 48 hours** |  |  |  |
| Low molecular weight heparin | 132 (46.8) | 44 (46.8) | 1.00 |
| Clopidogrel | 217 (77.0) | 77 (81.9) | 0.31 |
| GP IIb-IIIa inhibitors | 103 (36.5) | 33 (35.1) | 0.80 |
|  |  |  |  |
| **Procedures during hospital stay** |  |  |  |
| Coronary angiography | 223 (79.1) | 74 (78.7) | 0.94 |
| PCI | 164 (58.2) | 56 (59.6) | 0.81 |
| CABG | 8 (2.8) | 3 (3.2) | 0.86 |
|  |  |  |  |
| **In-hospital complications** |  |  |  |
| Reinfarction | 10 (3.5) | 3 (3.2) | 0.87 |
| Stroke | 6 (2.1) | 2 (2.1) | 1.00 |
| Major bleeding | 6 (2.1) | 7 (7.4) | 0.015 |
| Transfusion | 15 (5.3) | 13 (13.8) | 0.006 |
| Ventricular fibrillation | 4 (1.4) | 12 (12.8) | <0.001 |
| Atrial fibrillation (new) | 20 (7.1) | 24 (25.5) | <0.001 |
| AV block | 9 (3.2) | 4 (4.3) | 0.62 |
|  |  |  |  |
| **Medications at discharge** |  |  |  |
| Aspirin | 248 (87.9) | 85 (90.4) | 0.51 |
| Clopidogrel | 207 (73.9) | 75 (80.6) | 0.19 |
| Statin | 209 (74.1) | 68 (72.3) | 0.73 |
| Beta-blocker | 199 (71.3) | 58 (63.7) | 0.17 |
| ACE-inhibitor | 188 (67.4) | 64 (68.8) | 0.80 |
| ARB | 21 (7.8) | 4 (4.4) | 0.28 |
| Aldosterone receptor blocker | 17 (6.3) | 17 (18.7) | <0.001 |
| Loop diuretic | 79 (29.8) | 51 (56.7) | <0.001 |
| Digoxin | 2 (0.7) | 0 | 0.41 |
| Nitrates | 55 (20.4) | 25 (27.8) | 0.15 |
| Amiodarone | 26 (9.2) | 23 (24.5) | <0.001 |

**Abbreviations:** ACE: angiotensin converting enzyme; ARB: angiotensin receptor blockers; AV: atrio-ventricular; BMI: body mass index; CABG: coronary artery bypass graft; CAD: coronary artery disease; COPD: chronic obstructive pulmonary disease; LVEF left ventricular ejection fraction; PCI: percutaneous coronary intervention; STEMI: ST-segment elevation myocardial infarction.
