# Supplementary material for: Long-term outcome in early survivors of cardiogenic shock at the acute stage of myocardial infarction: a landmark analysis from the French registry of Acute ST-elevation and non-ST-elevation Myocardial Infarction (FAST-MI) Registry
Source: Crit Care. 2014 Sep 19;18(5):516. doi: 10.1186/s13054-014-0516-y (PMC4192440; doi:10.1186/s13054-014-0516-y)
Supplement: Additional file 3: — Propensity-score-matched cohorts of patients alive at one year. [file 13054_2014_516_MOESM3_ESM.doc]

**Additional file 3: Table S3: Propensity score-matched cohorts of patients alive at one year**

|  | **No shock**  **N=217** | **Shock**  **N=73** | **P value** |
| --- | --- | --- | --- |
| Age (years), mean ± SD | 67.6 ± 12.8 | 68.1 ± 13.1 | 0.79 |
| Sex (F) | 80 (36.2) | 24 (32.9) | 0.54 |
| BMI (Kg/m²) mean ± SD | 27.3 ± 4.4 | 27.0 ± 4.8 | 0.61 |
|  |  |  |  |
| **Risk factors** |  |  |  |
| Hypertension | 112 (51.6) | 40 (54.8) | 0.64 |
| Diabetes mellitus | 66 (30.4) | 23 (31.5) | 0.86 |
| Current smoking | 64 (29.5) | 21 (28.8) | 0.91 |
| Hypercholesterolemia | 112 (51.6) | 35 (47.9) | 0.59 |
| Family history of CAD | 16 (7.4) | 7 (9.6) | 0.54 |
|  |  |  |  |
| **Previous medical history** |  |  |  |
| Myocardial infarction | 52 (24.0) | 19 (26.0) | 0.72 |
| PCI | 22 (10.1) | 9 (12.3) | 0.60 |
| CABG | 15 (6.9) | 6 (8.2) | 0.71 |
| Stroke | 6 (2.8) | 3 (4.1) | 0.57 |
| Peripheral arterial disease | 27 (12.4) | 10 (13.7) | 0.78 |
| Heart failure | 22 (10.1) | 7 (9.6) | 0.89 |
| Chronic kidney disease | 5 (2.3) | 2 (2.7) | 0.83 |
| COPD | 21 (9.7) | 6 (8.2) | 0.71 |
| Cancer | 1 (0.5) | 2 (2.7) | 0.10 |
|  |  |  |  |
| **Previous medications** |  |  |  |
| Antiplatelet agents | 57 (26.3) | 22 (30.1) | 0.52 |
| Statins | 66 (30.4) | 25 (34.2) | 0.54 |
| ACE-inhibitors | 64 (29.5) | 23 (31.5) | 0.74 |
| ARBs | 34 (15.7) | 10 (13.7) | 0.68 |
| Beta-blockers | 49 (22.6) | 18 (24.7) | 0.72 |
| Insulin | 23 (10.6) | 8 (11.0) | 0.93 |
|  |  |  |  |
| **Current episode** |  |  |  |
| Typical chest pain | 158 (75.6) | 46 (69.7) | 0.34 |
| Resuscitated cardiac arrest | 1 (0.5) | 8 (11.0) | <0.001 |
| ST-elevation MI | 120 (55.3) | 37 (50.7) | 0.49 |
| Anemia on admission | 46 (21.9) | 19 (27.5) | 0.34 |
| Admission glycemia (mg/dl) mean ± SD | 156 ± 70 | 184 ± 99 | 0.01 |
| LVEF (%) mean ± SD | 50.1 ± 12.5 | 43.9 ± 16.3 | 0.002 |
|  |  |  |  |
| **Medications within the first 48 hours** |  |  |  |
| Low molecular weight heparin | 117 (53.9) | 36 (49.3) | 0.50 |
| Clopidogrel | 180 (82.9) | 61 (83.6) | 0.90 |
| GP IIb-IIIa inhibitors | 72 (33.2) | 27 (37.0) | 0.55 |
|  |  |  |  |
| **Procedures during hospital stay** |  |  |  |
| Coronary angiography | 179 (82.5) | 60 (82.2) | 0.95 |
| PCI | 127 (58.5) | 46 (63.0) | 0.50 |
| CABG | 12 (5.5) | 3 (4.1) | 0.63 |
|  |  |  |  |
| **In-hospital complications** |  |  |  |
| Reinfarction | 4 (1.8) | 2 (2.7) | 0.64 |
| Stroke | 3 (1.4) | 2 (2.7) | 0.44 |
| Major bleeding | 2 (0.9) | 6 (8.2) | 0.001 |
| Transfusion | 6 (2.8) | 9 (12.3) | 0.001 |
| Ventricular fibrillation | 4 (1.8) | 11 (15.1) | <0.001 |
| Atrial fibrillation (new) | 7 (3.2) | 17 (23.3) | <0.001 |
| AV block | 3 (1.4) | 3 (4.1) | 0.17 |
|  |  |  |  |
| **Medications at discharge** |  |  |  |
| Aspirin | 192 (88.5) | 66 (90.4) | 0.65 |
| Clopidogrel | 161 (74.2) | 57 (79.2) | 0.40 |
| Statin | 163 (75.1) | 57 (78.1) | 0.61 |
| Beta-blocker | 163 (75.8) | 47 (67.1) | 0.15 |
| ACE-inhibitor | 137 (63.1) | 54 (75.0) | 0.065 |
| ARB | 21 (10.0) | 2 (2.9) | 0.06 |
| Aldosterone receptor blocker | 15 (7.1) | 14 (20.0) | 0.002 |
| Loop diuretic | 51 (24.5) | 38 (55.1) | <0.001 |
| Digoxin | 3 (1.4) | 0 | 0.32 |
| Nitrates | 33 (15.7) | 16 (23.2) | 0.16 |
| Amiodarone | 19 (8.8) | 18 (24.7) | <0.001 |
|  |  |  |  |
| **Medications at one year** | n=160 | n=55 |  |
| Aspirin | 132 (82.5) | 45 (81.8) | 0.91 |
| Clopidogrel | 115 (71.9) | 37 (67.3) | 0.52 |
| Statin | 128 (80.0) | 45 (81.8) | 0.77 |
| Beta-blocker | 116 (72.5) | 44 (80.0) | 0.27 |
| ACE-inhibitor | 95 (59.4) | 35 (63.6) | 0.58 |
| ARB | 32 (20.0) | 12 (21.8) | 0.77 |
| Aldosterone receptor blocker | 15 (9.4) | 10 (18.2) | 0.08 |

**Abbreviations:** ACE: angiotensin converting enzyme; ARB: angiotensin receptor blockers; AV: atrio-ventricular; BMI: body mass index; CABG: coronary artery bypass graft; CAD: coronary artery disease ; COPD: chronic obstructive pulmonary disease; LVEF left ventricular ejection fraction; PCI: percutaneous coronary intervention; STEMI: ST-segment elevation myocardial infarction.
