# Supplementary material for: Long-term outcome in early survivors of cardiogenic shock at the acute stage of myocardial infarction: a landmark analysis from the French registry of Acute ST-elevation and non-ST-elevation Myocardial Infarction (FAST-MI) Registry
Source: Crit Care. 2014 Sep 19;18(5):516. doi: 10.1186/s13054-014-0516-y (PMC4192440; doi:10.1186/s13054-014-0516-y)
Supplement: Additional file 4: — Characteristics of patients in the propensity-score-matched cohorts of 1-year survivors according to survival status at 5 years. [file 13054_2014_516_MOESM4_ESM.doc]

**Additional file 4: Table S4:** Characteristics of patients in the propensity-score-matched cohorts of one-year survivors according to survival status at 5 years

|  | **No cardiogenic shock (n=217)** | | **Cardiogenic shock (n=73)** | |
| --- | --- | --- | --- | --- |
|  | Alive  at 5 years | Dead  at 5 years | Alive  at 5 years | Dead  at 5 years |
| Age (years) | 64 ± 12 | 75 ± 10 | 75 ± 10 | 79 ± 6 |
| Sex (% F) | 37 | 37 | 33 | 33 |
| LVEF (%) | 50 ± 12 | 46 ± 11 | 46 ± 16 | 36 ± 13 |
| Hypertension (%) | 44 | 78 | 49 | 72 |
| Diabetes (%) | 23 | 57 | 27 | 44 |
| Prior myocardial infarction (%) | 17 | 47 | 16 | 56 |
| History of heart failure (%) | 4 | 33 | 7 | 17 |
| STEMI (%) | 64 | 26.5 | 67 | 0 |
| LMWH use (%) | 59.5 | 35 | 53 | 39 |
| Clopidogrel use (%) | 91 | 80 | 91 | 61 |
| Coronary angiography (%) | 89 | 61 | 96 | 39 |
| PCI (%) | 65.5 | 35 | 78 | 17 |

**Abbreviations:** LMWH Low molecular weight heparin; LVEF left ventricular ejection fraction; PCI: percutaneous coronary intervention; STEMI: ST-segment elevation myocardial infarction.
